# Electricity in Gynecology

**Published:** 1889-08

**Authors:** W. E. B. Davis

**Affiliations:** Birmingham, Ala.


### ELECTRICITY IN GYNECOLOGY.

AN ABSTRACT OF A PAPER READ AT THE RECENT MEETING OF THE ALABAMA MEDICAL ASSOCIATION.

BY DR. W. E. B. DAVIS, OF BIRMINGHAM.

He believes that ultra-enthusiasm has led to frequent failure in the use of this remedy, but says there should be no question as to its importance as a therapeutic agent in gynecological practice when such men as Apostoli, the Keiths, Engelmann and other competent observers, who have had experience in its application, report most satisfactory results. His apparatus consists of a Gaiffe faradic battery; the bi-polar uterine and vaginal excitor of Apostoli; a fifty-cell galvanic battery, constructed by Woodruff & Harris, of Birmingham, under his supervision, the cells of which are of the Law telephone pattern; a portable Waite & Bartlett galvanic battery; Gaiffe's galvanometer; Massey's current controller; the abdominal electrodes of Apostoli and of Martin; platinum sounds, steel needles; and metal electrodes to be used with absorbent cotton.

He advises the use of the current of the Edison circuit, direct from the dynamo, when it can be had, and thereby avoid the annoyances and inconveniences of a battery. Portable batteries have proved very disappointing for the administration of high intensities, and his work has been confined principally to office practice. Great stress is laid on the importance of the application of the faradic current in sub-involution of the uterus, and every woman who has had an abortion or is confined at full term, is placed on ergot, and should there be incomplete involution at the expiration of six weeks, he begins at once the use of the faradic current, with the bi-polar, intra-uterine excitor of Apostoli, and repeats the application every second or third day until the organ has returned to its normal size, "which can always be counted on with mathematical certainty." He does not recommend the use of the current immediately after every abortion or delivery, as practiced by Apostoli, since this treatment could not or would not be afforded, except by a very small class, unless it were certain that the uterus would not return to its proper size. For this reason ergot is prescribed in every case, as stated, since it acts very much as faradization on the smooth, non-striated muscular fiber of the uterus, although not by any means so prompt, energetic and reliable. All cases are examined at the expiration of six weeks to ascertain whether involution has been complete.

Cases are reported to show the value of the faradic current in sub-involution of the uterus, and to illustrate its efficacy in displacements due to the enlarged, hyperæmic condition of the organ following parturition. The current of tension, the current from the long, fine wire, has proven a valuable agent for the relief of pain, and cases showing permanent relief were quoted.

To illustrate the power of the faradic current on the perineum, vagina and uterine ligaments in prolapsus of the uterus, and also the effects of the current in relieving pain, the following case was reported:

Mrs. P., aged 35 years; had last child four years ago; since which time she had suffered almost all the time from pelvic pains, insomnia and very marked nervous symptoms. The pain was so severe at her menstrual periods that she had been advised by her physician to have her ovaries removed, and it was for this purpose that he was consulted. Her uterus was normal in size and prolapsed to a marked degree, which was due to its relaxed supports. The bi-polar vaginal excitor, with strong current, was used every second or third day for two weeks, and at the next period she did not know when the flow appeared. In eight weeks her uterus remained in proper position; she was relieved of insomnia and her nervous symptoms improved.

Two months after the treatment was stopped she became pregnant.

Both the currents of quantity and tension were used at each consultation in this case—the former for the relaxed muscle, the latter for pain. The patient always felt better after each seance.

The currents of quantity and tension have been used with very satisfactory results, as indicated by Apostoli, but he has begun to use the current of tension not only for pain, but to stimulate relaxed and enfeebled muscle fiber. The current of tension is borne better by the patient, and he has been unable to recognize the superior results of the current of quantity on muscle over the current tension. In displacements of the uterus, he supports the organ with wool tampons, and does not object to any form of pessary, properly fitted, in connection with the treatment by electricity. He believes proper support of the organ, combined with the proper application of electricity, to be the most rational treatment for this condition.

When the uterus is enlarged not from sub-involution, but *hyperplasia*, the continuous current is indicated. All cases of chronic endometritis are amenable to galvanism*—the positive current when there is much leucorrhœa or profuse menstruation, and the negative in other cases. From seventy-five to one hundred and fifty milliamperes are used twice weekly, for five minutes at a time. The sound is usually introduced through a bivalve speculum, and the handle allowed to rest on a large wad of absorbent cotton, which prevents injury to the endometrium. This is preferred because it permits of more thorough antisepsis,

* Apostoli, Chronic Metritis, etc.

and allows the physician to rest his hand during the operation. He does not say that electricity will do away entirely with such surgical procedures as shortening the round ligaments—Alexander's operation, or attaching the cornua of the organ to the abdominal wall, or the narrowing of the vagina by the many methods at present in vogue, but he insists that many cases can be relieved by this method of treatment, which would otherwise be condemned to the knife.

Chronic inflammatory exudations in the pelvis should be punctured from once to twice a week, and from one hundred to one hundred and fifty milliamperes of the negative current used. The faradic current is an admirable remedy for the so-called chronic pelvic inflammation—thickening of one or both broad ligaments from the collection of blood in the distended veins when the uterus is displaced.† Of course the lacerated cervix which usually causes this condition, should be repaired before the administration of electricity is begun.

The local application of the faradic current is capable of relieving many cases of amenorrhœa, due to atrophy of the uterus. In menorrhagia, due to relaxation of muscle, to engorgement, when patient menstruates from eight to nine days; after a few applications, the menstrual periods would only last from four to five days. The positive galvanic current is the remedy indicated for hemorrhage due to a disease of the endometrium, and is the current usually indicated for hemorrhage. Women often become pregnant soon after being treated by electricity, and it is unquestionably a valuable remedy for sterility due to nervous causes, so ably described by Dr. Campbell.

Neuralgic dysmenorrhœa and dysmenorrhœa in women of a hysterical temperament, whom the slightest excitement or worry will cause to suffer greatly, those cases where there is no apparent pathological lesion, he has succeeded, as with no other remedy, by the application of the current of tension, or by the mild, positive, galvanic current.

The negative current is indicated when the pain is due to me-

---

† Hardon, Transactions S. S. & G. Association.

chanical causes in the cervical canal, and when there are inflammatory deposits around the ovaries, etc.

He does not think that galvanism can take the place of the removal of the ovaries and tubes, but says each has its special field, and should electricity fail, there is no harm done, and the operation can still be resorted to.

While he has had no experience with electricity in extra-uterine pregnancy, from a study of the actions of the agent and the results in the hands of others, thinks there can be no doubt but what it should be used in the early stages of this condition, and should there be a mistake in diagnosis, there could be no harm done, as this is the remedy for the pathological processes which are liable to be mistaken for extra-uterine gestation. When the pregnancy has lasted for more than three months, and when it can be positively diagnosed, it is a question in his mind whether laparotomy should not be resorted to at once.

He said the subject which had concerned the profession most in connection with the use of electricity was *the treatment of fibroid tumors*, and that the results of the treatment in the hands of Apostoli, the Keiths, Engelmann, Lapthorn Smith and others, had demostrated that this is *the treatment* for fibroid tumors, which "offer probabilities of healthy retrograde metamorphosis." —*Engelmann.*

He had followed Apostoli's instructions in this class of neoplasms, and believed that the majority of cases could be symptomically cured. Certainly Apostoli's treatment should be tried before resorting to hysterectomy.

